# The 500-year Cultural & Economic Trajectory of Tobacco: A Circle Complete

**DOI:** 10.36469/9809

**Published:** 2017-12-20

**Authors:** Christopher A. Jones, Amanda Wassel, William Mierse, E. Scott Sills

**Affiliations:** 1 Department of Biomedical Informatics, College of Health Solutions; University of Vermont Health Network & Socio-Ecological Gaming Simulation Laboratory of the Community Development and Applied Economics Department; Arizona State University, Phoenix, Arizona, USA; University of Vermont, Burlington, Vermont, USA; European Centre for International Political Economy (ECIPE), Brussels, Belgium; 2 University of Vermont Health Network & Socio-Ecological Gaming Simulation Laboratory of the Community Development and Applied Economics Department University of Vermont, Burlington, Vermont, USA; 3 Department of History and Art History University of Vermont, Burlington, Vermont, USA; 4 Department of Obstetrics & Gynecology Center for Advanced Genetics; Carlsbad, California, USA; Palomar Medical Center, Escondido, California USA

**Keywords:** smoking, regulation, religion, society, health economics, economics, rewards, incentives, tobacco

## Abstract

Who smokes, and why do they do it? What factors discourage and otherwise reward or incentivize smoking? Tobacco use has been accompanied by controversy from the moment of its entry into European culture, and conflicting opinions regarding its potentially adverse influence on health have coexisted for hundreds of years. Its use in all forms represents the world’s single greatest cause of preventable disease and death. Tobacco was introduced to Europe by Christopher Columbus, who in October 1492 discovered the crop in Cuba. While the next four centuries would see tobacco as the most highly traded economic commodity, by 1900, the now familiar cigarette remained obscure and accounted for only 2% of total tobacco sales. Global tobacco consumption rose sharply after 1914 and became especially prevalent following World War II, particularly among men. Indeed, overall tobacco sales increased by more than 60% by the mid-20th century, and cigarettes were a critical driver of this growth. Cigarettes dominated the tobacco market by 1950, by then accounting for more than 80% of all tobacco purchases. In the absence of clinical and scientific evidence against tobacco, moral and religious arguments dominated opposition voices against tobacco consumption in the 1800s. However, by the mid-20th century, advancements in medical research supported enhanced government and voluntary actions against tobacco advertising and also raised awareness of the dangers associated with passive tobacco smoke exposure. Solid epidemiological work connecting tobacco use with “the shortening of life span” began to appear in the medical literature in the 1950s, linking smoking with lung cancer and related conditions. In subsequent years, these developments led to significant curtailment of tobacco use. This monograph explores aspects of the intersection of tobacco with themes of behavioral incentives, religion, culture, literature, economics, and government over the past five centuries.

## INTRODUCTION

A century-old riddle sets a peculiar challenge:

*To three fourths of a cross add a circle complete;*
*Let two semicircles a perpendicular meet;*
*Next add a triangle that stands on two feet;*
*Then two semicircles, and a circle complete.*

Only one word in English could spell the answer to this geometry-savvy puzzle, as first framed in the 1907 George Welsh novel *The Fragrant Weed*. Cryptically packaging the familiar subject of tobacco as a playful riddle was not without good economic reason. Tobacco had already been the world's single largest revenue-creating commodity for centuries. Long before Columbus set sail on the *Santa Maria*, this lant was used by native peoples for trade and in ritual in the Americas, and its popularity quickly spread after its introduction into Europe. Columbus himself described natives of the West Indies burning "perfumed herbs" and intentionally breathing the resulting smoke.[1]

Between 1492 and 1660, European explorers across the Americas recorded some 50 distinct native populations who used the tobacco plant. These foreign observers reported that tobacco was used for religious ceremonies and medicinal purposes, such as for toothaches, eye problems, and wounds.[1] In the 1530s, during his travels near modern-day Montreal, Jacques Cartier described men carrying dried tobacco in bags and using pipes to "fill their bodies full of smoke, till that it commeth out of their mouth and nostrils, even as out of the tonnel of a chimny." Tobacco use was not limited to the North American continent; the first protestant missionary in Brazil in the mid-1550s, Jean de Lery, sampled the tobacco of the local inhabitants and wrote that, "it seemed to satisfy and ward off hunger."[1]

The phenomenal growth of interest in tobacco resulted from a lethal combination of addiction and profitability—factors which transformed tobacco into a cash crop on a scale scarcely seen before or since in world history. Yet, with tobacco's immense popularity have come health consequences of an equally astounding magnitude. From a brain science perspective, tobacco is known to alter the hypothalamic-pituitary axis that controls fertility in addition to important growth and development functions. Tobacco is known to cause growth retardation and delayed brain development in infants born to mothers who smoke during pregnancy. Tobacco also predisposes populations to an increased likelihood and severity of costly chronic disease. We are well aware of the dangers of smoking. We may be less aware that the arc of tobacco's cultural reception has been for centuries modulated by religion and government, tracing an unusual trajectory. In this work, we explore how these forces helped shape the modern cultural understanding of tobacco.

## "PHYSIC" AND EARLY ADVOCACY FOR TOBACCO

Tobacco entered the European marketplace around 1574 in part with the help of a popular medical text by Nicolas Monardes. This publication (and others of similar type) attempted to establish tobacco as a medical panacea. Disguised as a useful tool in contemporary medical practice, tobacco benefitted from an early association with professional medical practice and was robustly welcomed. While early shipment records (c.1600) show that some 25 000 pounds of tobacco were being imported into England every year, as the 17$^{th}$ century closed, this figure had increased to around 38 000 000 pounds annually.[1] Although tobacco was warmly extolled as an essential cure-all by physicians and traders, there were contemporary detractors who voiced clear opposition to it. For example, King James I strongly denounced tobacco in his 1604 pamphlet

"*A Counterblaste to Tobacco*" which condemned the plant both for its negative spiritual and health effects. Smoking tobacco, James declared, was "sinning against God… [It was a] custome loathsome to the eye, hatefull to the nose, harmefull to the braine, dangerous to the lungs, and in the blacke stinking fume thereof, nearest resembling the horrible stigian smoke of the pit that is bottomelesse."[2]

Yet such powerful statements by James I and other non-physicians had little effect on reducing the grip of tobacco upon the English people, and even less on the world beyond his realm. Up to the present day, tobacco has maintained a deadly hold on generations of men and women in every nation. As a commodity, it has funded wars, empires, expeditions, and helped build the very fabric of global economies. As accessory baggage on the worldwide tobacco train, numerous communicable diseases (polio, influenza, smallpox, syphilis, etc.) coming into and out of the "New World" brought millions to an early demise.[1] Although cures for these ailments did eventually come in the form of medicines, vaccines, antibiotics, and better standards of living, human mortality curves shifted and unmasked lung cancer and other tobacco-related health problems as an important scourge upon human health for many generations.

## ECONOMIC & DEMOGRAPHIC DIMENSIONS

In the United States, more than 16 million individuals now live with a disease caused by tobacco use including cancer, heart disease, and chronic obstructive pulmonary disease.[3] The total annual economic impact of smoking in the United States in 2014 included about $170 billion in direct medical costs and about $156 billion in lost productivity.[4] In the early 20$^{th}$ century, governments did little to discourage smoking. During World War II and the Korean Conflict, the U.S. Government provided no-cost cigarette allowances to soldiers stationed overseas while sharply discounting cigarette prices on U.S. military bases.[5] In 1993, the U.S. Department of Veterans Affairs officially acknowledged the link between their past tobacco policies and negative health outcomes. As late as 2006, the U.S. Defense Department subsidized tobacco products for service members despite the U.S. Surgeon General's report more than twenty years earlier detailing the dangers of smoking.[5] The tobacco companies countered with the hollow innovation of "low tar" products and modified cigarette filter designs—marketing measures which had the unintended effect of attracting more women and minorities as first time smokers.

In 1984, the U.S. Congress passed the Comprehensive Smoking Education Act which mandated changes in the way health warnings were displayed on cigarette packaging, including the statement "*Smoking by pregnant women may result in fetal injury, premature birth, and low birth weight.*" But have these anti-smoking measures worked? The results of this public health initiative, unfortunately, have been mixed: effectiveness in consumer education in the United States is likely positive, although the impact on tobacco consumption worldwide remains closely tied to monetary interests and is decidedly less encouraging.

For example, in 2009 the highest percentage of male smokers over the age of 15 could be found in the Pacific island nation of Kiribati (more than 70%), followed by Greece at 63% and Indonesia at 61%. Likewise, the highest percentage of female smokers over age 15 was in Nauru (50%), followed by Austria (45%) and Kiribati (43%).[6,7] Notably, China alone had >350 million smokers in 2003; their state-owned tobacco company manufactured 1.7 trillion cigarettes generating a profit of some two billion U.S. dollars—contributing 7.4% of central government revenue in China.[8] While the Chinese government is aware of the negative health effects of tobacco and has taken official steps to restrict cigarette advertisements, reduce teen smoking, and discourage smoking in public, the sale of tobacco products curiously remains untaxed in China.[8]

India has the second largest population of smokers in the world. Indian health data from 2004 show that the direct medical costs to treat four major tobacco-related diseases was $1.2 billion, consuming 4.7% of the country's total healthcare budget. Every year, about one million deaths occur in India due to tobacco-related diseases.[9]

Tobacco use thus poses an interesting economic dilemma that has remained unsettled for many years. While the individual and societal health costs of tobacco are undeniably high, the combined forces of corporate profits (including the impact of tobacco tax revenues and related lobbying) and personal addiction make it impossible to reduce tobacco use merely to a supply and demand equation. According to one model, increasing taxes to raise the retail price of a standard pack of cigarettes by 42% would result in a 9% reduction in daily smoking (66 million fewer smokers and 15 million fewer smoking-attributable deaths among adults in 2014). Revenue derived from tobacco sales would increase by 47%, yielding an additional $190 billion for government health spend.10 Against this background, more recent tactics of incentives against (or disincentives for) smoking have emerged with most jurisdictions requiring warnings and images on cigarette packaging, state taxes imposed on purchases, and even clinical programs that offer incentives to palliate tobacco users through short-term goals, such as cash rewards offered for every week of pregnancy that is completed smoke-free.

## RELIGIOUS THOUGHT AND THE "TOBACCO CULTURE"

Even when not specifically crafted for public policy purposes, incentives may be used to promote positive behavior. This notion certainly preceded tobacco's arrival in European culture and famous examples survive in spiritual thought, art, and architecture. Among the most visually striking "incentives" are images developed in the 12th century as decoration for churches, particularly those on the pilgrimage route to Santiago de Compostela. The Last Judgement was a common theme in these depictions, and that at Saint Lazare at Autun by Gislebertus offers perhaps a representative illustration. It occupies the arched space (tympanum) above the structure's main entrance. The central figure is Christ the Judge presented as isolated from the rest of the scene, impassive and uninterested in what happens around him. He of course needs no incentive, but appears as one. To his right are the saved, to his left the damned, and below are seen souls awaiting the final judgment. The immediate message seems apparent, "*At the moment of the Last Judgment, it will be too late to repent*." Those who have lived a good life will join the ranks of the saved, while those who have not repented will face the demons of hell. Monstrous forms hint of the pains that await the damned. Among the souls to be weighed are those of clergy and nobles, as well as those of more modest folk. All face the same fate, to be weighed and, if found wanting, to be condemned. The visual message is easy to read, and its intention is clear. To escape the horrors of hell, the soul must be judged to be pure. The behavior being promoted is that which corresponds to church teaching, and it is only by practicing such behavior that eternal damnation is to be avoided. Yet, Christ is shown as the highest incentive figure just as the demons and the hell represent disincentives. The message conveyed by this magnificent artwork can be summarized as: live a good Christian life, and you may spend eternity in heaven with your Savior.[11]

The Roman Catholic Church does not condemn smoking, but considers excessive smoking to be sinful, as described in the Catechism:[12] "The virtue of temperance disposes us to avoid every kind of excess: the abuse of food, alcohol, tobacco, or medicine." Jehovah's Witnesses have not permitted any active members to smoke since 1973, and their literature warns about physical and spiritual dangers of smoking.[13] These directives were later expanded to include electronic cigarettes.[14] Seventh-day Adventists, an international non-conformist denomination, have since their foundation in 1863, maintained a distinctive healthcare model for their members. The lifestyle has included vegetarian diet and abstinence from tobacco and other harmful substances.[15,16] Moreover, this religious group has called for governments to enact policies that include a uniform ban on all tobacco advertising, stricter laws prohibiting smoking in non-residential public places, more aggressive and systematic public education, and substantially higher taxes on cigarettes.[17]

The founder of the Latter Day Saint (LDS, Mormon) movement, Joseph Smith, recorded that on February 27, 1833 he received a revelation which addressed tobacco use. It is commonly known as the Word of Wisdom (Section 89 of the Doctrine and Covenants), and prohibits the smoking or chewing of tobacco. While initially regarded only as a guideline, this was eventually incorporated into official LDS Church policy and is now also observed to varying degrees by other LDS denominations. The effects of these practices have been extensively studied and the cancer rate (all types) for male LDS members is nearly 25% less than the comparable national rate. In addition, there appears to be a 50% lower rate of cancers associated with cigarette smoking among LDS men.[18]

There remains an extraordinarily high rate of tobacco use in southeast Asia.[19] Findings from national adult tobacco studies reveal that very few daily users of tobacco plan to discontinue its use. In predominantly Buddhist Cambodia, faith-based tobacco control programs have been implemented where, under the 5th Precept of Buddhism which proscribes addictive behaviors, monks were encouraged to quit tobacco and temples have been declared smoke-free. In one nationwide study on tobacco use throughout Cambodia, investigators found that most individuals (*n*=13 988) favored Buddhist monks not using any tobacco whatsoever and that the Wat (temple) should be smoke-free. Thus, anti-tobacco sentiments figure prominently in the belief system of Cambodian adults and could be helpful in augmenting anti-tobacco campaigns in this region.[20] Likewise in South America, religiosity has been identified as a strongly protective factor against tobacco consumption and use of other addictive substances among Brazilian university students.[21]

Islam was historically neutral towards smoking, but as health dangers emerged many leaders within influential mosques began to argue that it was "markrooh" (discouraged) or even "haram" (prohibited). Because such sentiments, if widely held, would be catastrophic to the tobacco economy as well as to collective human addictions, Islamic scholars sympathetic to tobacco have been enlisted to argue against strict prohibitions; Islamic theology has also been combed to provide interpretations of the Koran that are friendlier to tobacco. "The industry has sought to distort and misinterpret the cultural beliefs of these communities and to reinterpret them to serve the industry's interests," noted Kelley Lee of Simon Fraser University, one of the authors involved in researching this topic. "All to sell a product that kills half of its customers."[22]

Populations with large Islamic influences remain critically important to transnational tobacco corporations because they represent growth markets where smoking rates are likely to rise. In contrast, recruitment of new smokers in the Middle East has been less effective among Jewish males, where tobacco uptake has been relatively low for several years.[23] In parallel, corporate interests perceive Islam as developmentally antagonistic to tobacco markets where demographic features offer relatively large and young populations of millions of potential future customers. To maximize penetration into these emerging markets, the tobacco industry has mobilized alliances to negate any regulation hostile to its commercial interests,[24,25] and began to frame antismoking views within the Islamic community as eccentric and fanatical.26 Not surprisingly, such efforts to find culturally appropriate measures to support tobacco use conflict with the provisions of the World Health Organization's Framework Convention on Tobacco Control.[26]

Evidence exists to support the association of positive cultural and artistic smoking portrayals with youth smoking initiation, thus captivating new tobacco consumers who are likely to purchase the product for decades. In Iran, the ten most commercially successful films released over the past 30 years were recently assessed to measure this effect. The proportion of Persian movies depicting tobacco use was 36% in 1982-1991, 60% in 1992-2001 and 74% in 2002-2011, and the mean proportion of each movie's running time where smoking was shown also grew significantly during this period.[27] Perhaps more alarmingly, while the depiction of tobacco use in American (G-rated) films seems to be decreasing, parents should be aware that nearly half of feature "films for children" normalize tobacco use and fail to convey its long-term health effects to impressionable audiences.[28] In social media, researchers (including author CJ) found that 80% of e-cigarette related tweets in the Twittersphere were found to be "bots," created by e-cigarette companies, targeting kids and purporting to be other kids sharing their "success" stories with e-cigarettes.[29]

## CONCLUSIONS

The most recent information regarding worldwide smoking shows that about 80% of smokers are men and many of them live in developing countries. Tobacco remains very big business, no matter what scale is used—nearly 20% of the world population now smokes cigarettes. While the prevalence of tobacco use has plateaued or diminished in countries with advanced economies, the rest of the world shows an uneven consumption pattern with only 10 countries accounting for 60% of all tobacco use worldwide—a list led by China. Many interlocking cultural and economic factors are responsible for this arrangement.

Tobacco markets have not been financially satisfying in some locations. In Australia, smoking is in decline with 2011–13 data revealing only 16% of the adult population smokes—down from a rate of 22.4% in the previous decade. In the USA, smoking rates have fallen precipitously over 40 years, from 42% to about 20% of adults (1965 vs. 2006). When adequately resourced, the smoking cessation efforts for many states have shown a nearly immediate return on investment (ROI), with every 1 USD invested achieving 3-5 USD in downstream savings, especially in the area of maternal-fetal medicine, not to mention tobacco as a gateway drug to many other substances, including alcohol and opiates that have reached epidemic levels of misuse.

It is difficult to overstate the impact of socio-cultural elements of art and religion in fashioning health behaviors among young adults. Young adults are the most likely age group to take up smoking, with a marked decline in smoking rates as populations age, become better educated, and enter employment. Tobacco use is inversely associated with socioeconomic status, with the most disadvantaged quintile of the population registering a rate of tobacco consumption more than double that of the most socioeconomically advantaged "top quintile." Nowadays tobacco has evolved—its use in language has been replaced with terms like "e-cigarette." Smoking is part of our everyday culture; it is even a metaphor for describing beauty. Poet Laureate Donald Hall reminds readers of the "dead metaphor," and the circle complete has left more dead than all the combat and civilian casualties from all wars over the past century. Those least able to afford tobacco and its ill health consequences remain paradoxically the most likely to purchase tobacco products. It therefore appears that the old reservations concerning tobacco by James I were correct, after all; in this new century, we have indeed come "full circle." While our analysis merely scratches the surface of how interconnected tobacco is to history, economy, religion and culture, it is hoped that by highlighting these issues we may find better ways to navigate a healthier path on the circle of life.

## CONFLICT OF INTEREST

The authors report no conflict of interest.

## ACKNOWLEDGEMENTS

The authors wish to acknowledge the support of the Vermont Tobacco Evaluation and Review Board and Erin Hurley, Eric Clark and Rick Valenta for their helpful collaborations.

## References

[1] Mancall PC. Tales Tobacco Told in Sixteenth-Century Europe. *Env His* 2004;9(4):648-78.

[2] James I (of England). A Counterblaste to Tobacco (public domain). Univ Texas Library: https://www.laits.utexas.edu/poltheory/james/blaste/blaste.html. Accessed 29 August 2017.

[3] U.S. Surgeon General. The Health Consequences of Smoking—50 Years of Progress (Executive Summary). U.S. Department of Health & Human Services, Public Health Service, Office of the Surgeon General (Rockville, MD) 2014:1-36.

[4] Xu X, Bishop EE, Kennedy SM, Simpson SA, Pechacek TF. Annual healthcare spending attributable to cigarette smoking: an update. *Am J Prev Med* 2015;48(3):326-33.

[5] Bedard K, Deschênes O. The Long-Term Impact of Military Service on Health: Evidence from World War II and Korean War Veterans. *Am Econ Rev* 2006;96(1):176-94.

[6] World Health Organization Report on the Global Tobacco Epidemic, 2008. United Nations Press, *WHO* 2008: 8.

[7] World Health Organization Report on the Global Tobacco Epidemic, 2015. United Nations Information Office: http://www.who.int/tobacco/surveillance/policy/country_profile/kir.pdf Accessed 29 August 2017.

[8] Hu TW, Mao Z, Ong M, et al. China at the crossroads: the economics of tobacco and health. *Tob Control* 2006;15 Suppl 1:i37-41.

[9] John RM, Sung HY, Max WB, Ross H. Counting 15 million more poor in India, thanks to tobacco. *Tob Control* 2011;20(5):349-52.

[10] Goodchild M, Perucic AM, Nargis N. Modelling the impact of raising tobacco taxes on public health and finance. *Bull World Health Organ* 2016;94(4):250-7.

[11] Snyder J. Medieval Art: Painting-Sculpture-Architecture, 4th-14th Century. Prentice-Hall 1988:287 (Figs. 358 & 359).

[12] Vatican Library: Catechism of the Catholic Church (Part III, sec. 2). http://www.vatican.va/archive/ccc_css/archive/catechism/p3s2c2a5.htm. Accessed 29 August 2017.

[13] Watchtower [newspaper]. Awake! Why Quit Smoking? March 22, 2000:4-7.

[14] Watchtower [newspaper]. What Is God's View of Smoking? June 1, 2014:4.

[15] Eklöf M. Medicine on mission: The international health reform of Seventh-Day Adventists and their health care facilities in Sweden. *Sven Med Tidskr* 2008;12(1):119-41.

[16] McKenzie MM, Modeste NN, Marshak HH, Wilson C. Religious involvement and health-related behaviors among Black Seventh-Day Adventists in Canada. *Health Promot Pract* 2015 Mar;16(2):264-70.

[17] Seventh Day Adventist Statement. Smoking and Tobacco (policy directive). The Seventh-day Adventist Church: https://www.adventist.org/en/information/official-statements/statements/article/go/-/smoking-and-tobacco/ Accessed 29 August 2017.

[18] Lyon JL, Gardner K, Gress RE. Cancer incidence among Mormons and non-Mormons in Utah (United States) 1971-85. *Cancer Causes Control* 1994 Mar;5(2):149-56.

[19] Rerksuppaphol L, Rerksuppaphol S. Prevalence of Cigarette Smoking and Associated Risk Factors amongst Middle-School Students in Ongkharak District, Thailand. *J Med Assoc Thai* 2015;98 Suppl 9:S1-8.

[20] Yel D, Bui A, Job JS, Knutsen S, Singh PN. Beliefs about tobacco, health, and addiction among adults in Cambodia: findings from a national survey. *J Relig Health* 2013;52(3):904-14.

[21] Gomes FC, de Andrade AG, Izbicki R, Moreira Almeida A, Oliveira LG. Religion as a protective factor against drug use among Brazilian university students: a national survey. *Rev Bras Psiquiatr* 2013;35(1):29-37.

[22] Dyer O. Tobacco industry sought to prevent Islamic fatwas against smoking. *British Medical Journal* 2015;28(350):h2281.

[23] Saabneh AM. Arab-Jewish gap in life expectancy in Israel. *Eur J Public Health* 2015 Nov 26. pii: ckv211 (in press).

[24] Assunta M, Chapman S. A mire of highly subjective and ineffective voluntary guidelines: tobacco industry efforts to thwart tobacco control in Malaysia. *Tob Control* 2004;13 Suppl 2:ii43-50.

[25] Nakkash R, Lee K. The tobacco industry's thwarting of marketing restrictions and health warnings in Lebanon. *Tob Control* 2009;18(4):310-6.

[26] Petticrew M, Lee K, Ali H, Nakkash R. Fighting a hurricane: tobacco industry efforts to counter the perceived threat of Islam. *Am J Public Health* 2015;105(6):1086-93.

[27] Heydari G, Ebn Ahmady A, Lando HA, et al. Time trend of smoking scenes in Iranian movies during the past three decades (1982-2011): a historical analysis. *Tob Control* 2015 Jul 14. pii: tobaccocontrol-2014-051958 (in press).

[28] Thompson KM, Yokota F. Depiction of alcohol, tobacco, and other substances in G-rated animated feature films. *Pediatrics* 2001;107(6):1369-74.

[29] Clark EM, Jones CA, Williams JR, et al. Vaporous marketing: uncovering pervasive electronic cigarette advertisements on Twitter. *PLoS One* 2016; 11(7):e0157304.
